# Case Report: High-Definition 4K-3D Exoscope for Removal of an Orbital Cavernous Hemangioma Using a Transpalpebral Approach

**DOI:** 10.3389/fsurg.2021.671423

**Published:** 2021-08-06

**Authors:** Stefano Peron, Stefano Paulli, Roberto Stefini

**Affiliations:** ^1^Department of Neurosurgery, Azienda Socio Sanitaria Territoriale (ASST) Ovest Milanese - Legnano Hospital, Milan, Italy; ^2^Department of Maxillofacial Surgery, Azienda Socio Sanitaria Territoriale (ASST) Ovest Milanese - Legnano Hospital, Milan, Italy

**Keywords:** 4K-3D exoscope, navigation, orbital cavernous venous malformation, transpalpebral approach, ultrasound

## Abstract

**Background:** Cavernous hemangioma, also known as cavernous vascular malformation (CVM), is the most common primary lesion of the orbit in adults. The management of these lesions is challenging and is strongly dependent on their location, as well as the patient's symptoms and expectations. The trans-palpebral approach is currently used in surgery for orbital tumors, anterior skull base tumors, and even more, orbital reconstruction, because of its well-demonstrated esthetic advantages. Similarly, the use of magnification can be provided by surgical loupes, microscope, or more recently, endoscope, which is well-documented for its advantages in terms of minimal invasiveness and safety. In the last years, the use of exoscopes in microsurgery has been proposed due to their greater and sharper intraoperative magnification, but never for the removal of orbital tumors.

**Clinical Presentation:** We describe a case of a 38-year-old woman with a right orbital intraconic CVM removed using an inferior transpalpebral approach performed under 4K-3-dimensional (4K-3D) exoscopic vision. Navigation and ultrasound were also used, with the former allowing better identification of the lesion within the orbit and the second overcoming the limitations of navigation, in terms of the retraction on the ocular globe before or just after periorbital incision.

**Conclusion:** The use of a 4K-3D exoscope allowed us to perform the surgery safely, thanks to the high magnification and definition of anatomical details, with the surgeon operating in an upright, comfortable position. The CVM was completely removed with excellent results from both functional and esthetic points of view.

## Introduction

Cavernous venous malformation (CVM), also called cavernous hemangioma, is a benign, well-capsulated, vascular malformation, representing the most common primary orbital lesion of adults, with an incidence between 14.5 and 21.3% of all orbital tumors (1, 2). These lesions occur more often in women with clinical symptoms appearing typically in the fourth and fifth decades of life.

The most common sign of CVM is progressive axial proptosis due to the typical involvement of the intraconic orbital space. Optic nerve damage and other signs of orbital disturbance, such as visual impairment or diplopia, may be detected, but with a variable degree and frequency. The risk of orbital CVM rupture is very low, although, it is a described cause of non-traumatic retro-orbital hemorrhage.

Magnetic resonance imaging (MRI) of the orbits is the most important exam for correct diagnosis, with a combination of MRI, ultrasound, and computed tomography being accurate to precisely define the lesion in almost all cases (3).

Different surgical techniques have been proposed to remove these lesions, with lateral orbitotomy and anterior transconjunctival, or transpalpebral approaches as the most preferred for lateral CVMs, and an endoscopic endonasal approach is an effective and attractive alternative for medial CVMs (3–5).

Nonetheless, dealing with a small anatomical compartment with many delicate structures such as the orbit, all procedures for removal of intraconic lesions, either by trans-cranial or trans-palpebral approaches, require a magnified view of the anatomical details, which are traditionally provided by loupes or microscope.

In recent years, thanks to developments in surgical technology with procedures performed on head-up displays, exoscopes have also been introduced as an alternative or to assist microscopic vision.

To date, the use of the exoscope in surgery has been reported in the literature for cranial, spine, and otologic surgery (6–11). However, the use of a 4K-3D exoscopic system in a transpalpebral approach to the retro-orbital space has not yet been described.

## Case Presentation

A 38-year-old woman presented to the maxillofacial surgery clinic of the Department of Neurosciences at ASST Ovest Milanese complaining of mild proptosis of the right eye and minimal binocular diplopia in the right gaze, which had appeared a few months earlier.

**Figure 1:**
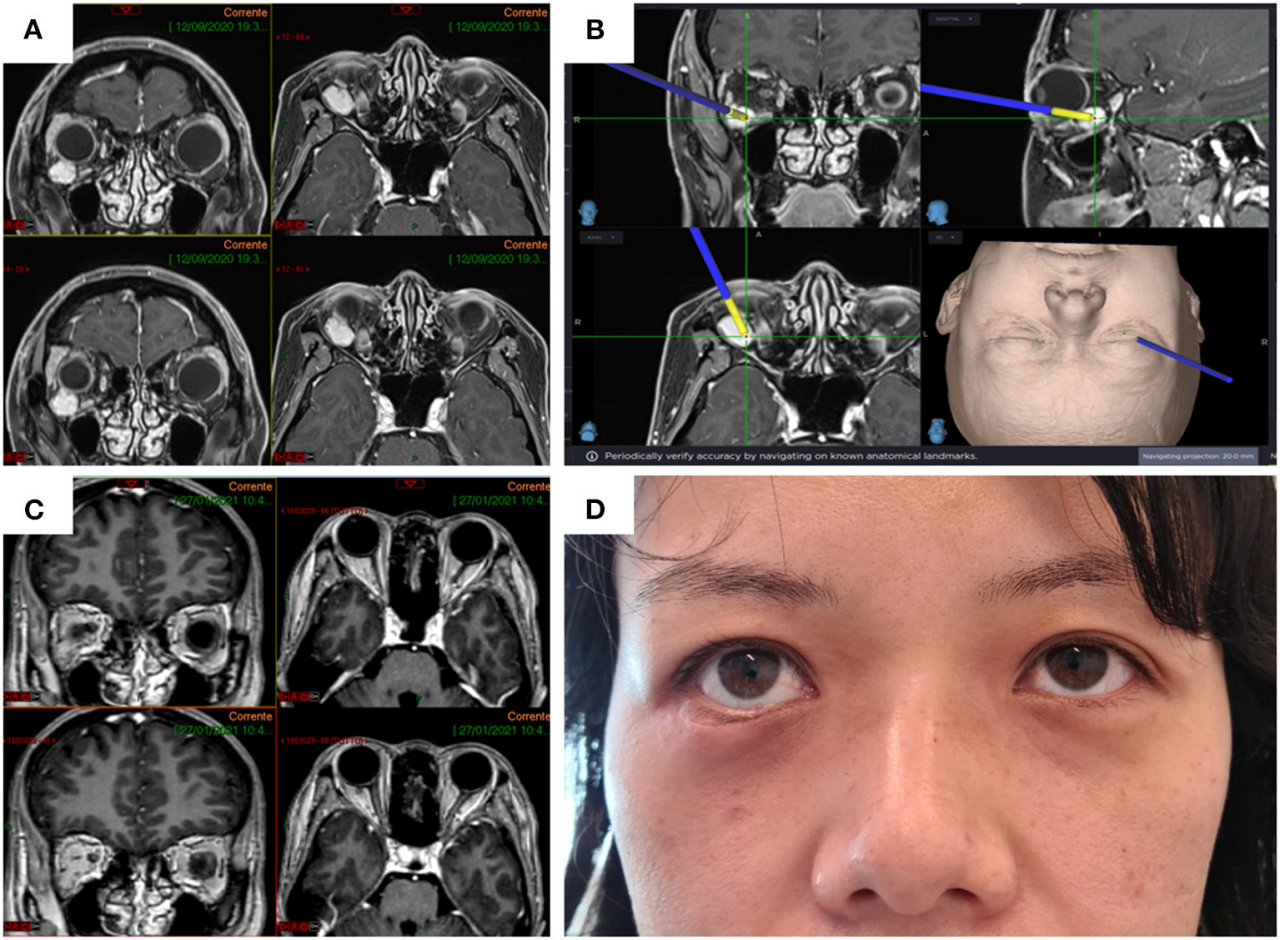
Preoperative enhanced MRI of the orbits (left, axial; right, coronal) showing the cavernous hemangioma located in the inferior lateral part of the right orbital intraconic space **(A)** and detail of tumor localization using navigation **(B)**. Postoperative MRI showing complete removal of the hemangioma **(C)**. The transpalpebral approach allows a minimally invasive access to the retro-orbital space ensuring excellent esthetic and functional results **(D)**.

MRI of the orbits showed an enhanced, T2-hyperintense, T1-isointense, intraconic lesion in the inferior lateral part of the right orbit just behind the ocular globe (Figures 1A,B).

Preoperatively, the patient underwent contrast-enhanced MRI with volumetric acquisition (1–1.20-mm slice thickness) for 3D reconstruction. The woman underwent surgery to remove the lesion with a transpalpebral approach through the right inferior eyelid. The surgical procedure was performed by the second author (S.P.), who has substantial experience in transpalpebral approaches for orbital tumors and orbital reconstruction.

The patient was placed in a supine position, with the head slightly rotated to the left side.

The entire surgical procedure, starting from the skin incision, was performed under 4K-3D exoscopic vision. After the skin incision of the lower eyelid, a blunt dissection to expose the inferior orbital rim up to the lateral canthus was performed, and then gently dissecting the periorbit from the lateral wall.

**Figure 2:**
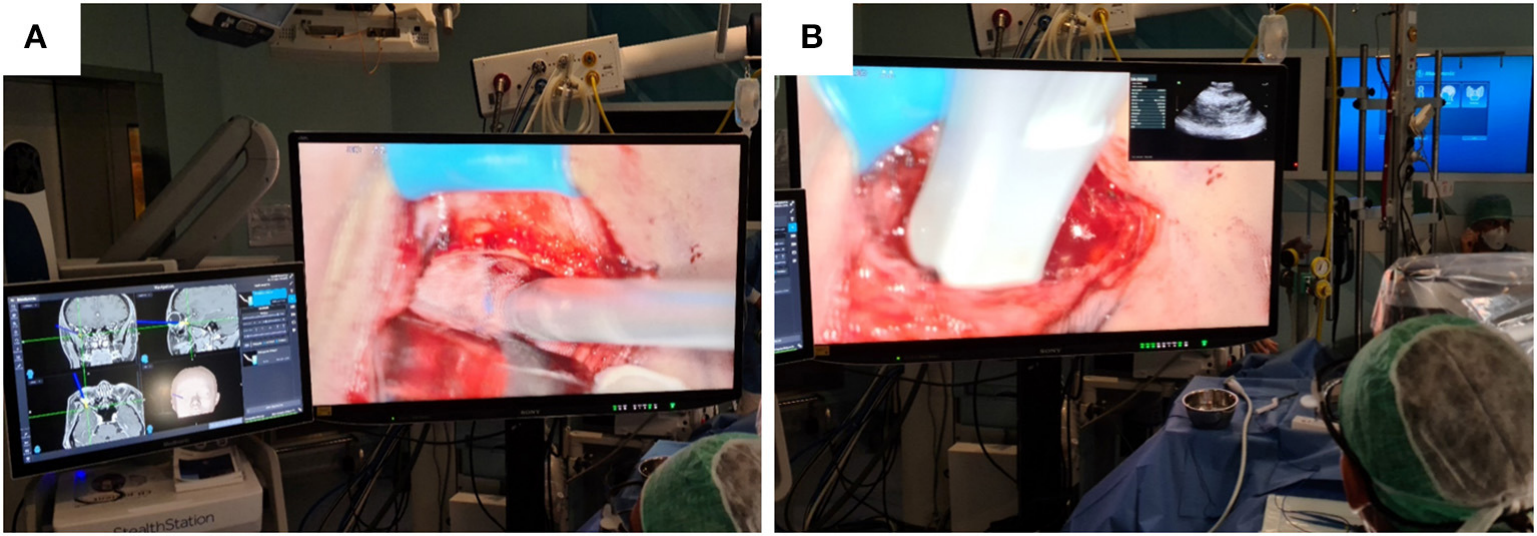
On the 55-inch monitor, the navigation device **(A)** and ultrasound probe **(B)** used for precise localization of the hemangioma inside the periorbita are clearly visible. The PiP mode **(B)** allows the surgeon to control the position of the ultrasound probe without having to look away from the operating field.

**Figure 3:**
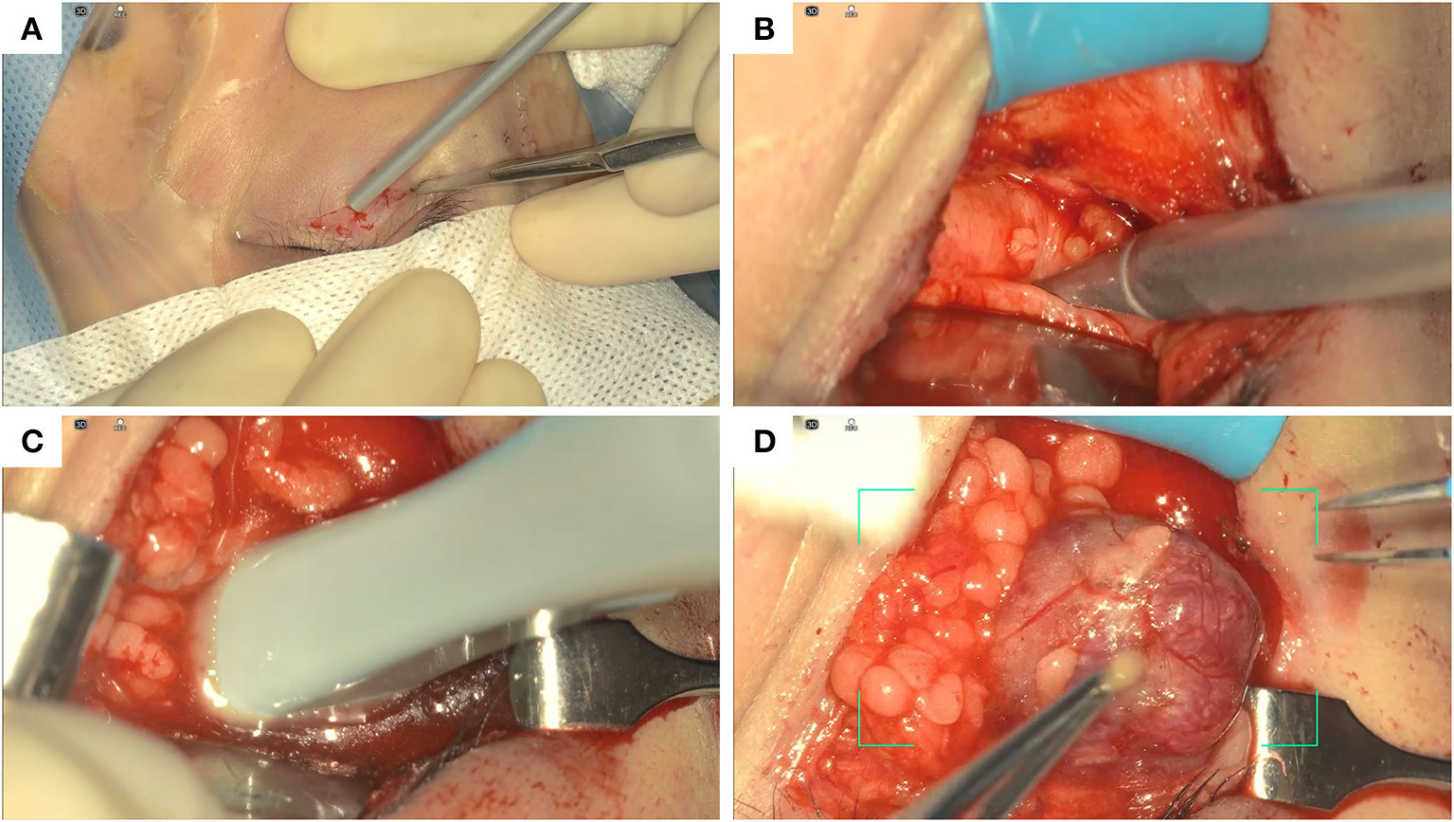
Summary of the main surgical steps. Skin incision of the lower eyelid **(A)**; localization of the hemangioma before the opening of the periorbit using navigation **(B)**; localization of the hemangioma within the periorbit using ultrasound **(C)**; blunt dissection and removal of the hemangioma **(D)**.

Minimally-invasive removal of the lesion was aided by the use of intraoperative navigation (electromagnetic StealthStation S8, Medtronic) and ultrasound (BK 5000®, BK Medical) in order to limit retraction on the ocular globe, and to reduce the incision of the periorbita and manipulation of intraconic structures within the periorbital fat (Figure 2). The main steps of the surgical procedure are clearly visible in the attached Video as well as summarized in Figure 3.

There were no complications during the procedure.

The surgical time was comparable to that of the same procedures previously performed by the same surgeon with loupes.

Postoperative MRI showed complete removal of the lesion (Figure 1C). The esthetic result for the patient was optimal (Figure 1D). Histological examination returned a diagnosis of cavernous hemangioma.

## Exoscope Characteristics

Our exoscope (ORBEYE, Sony Olympus Medical Solutions Inc., Tokyo, Japan) is equipped with an external 4K-3D orbital camera with a semi-robotic arm, which allows positioning above the operating field in multiple angles and a high optical zoom, with instant digital zoom for extra-detailed vision.

In addition to the camera, there are two medical 4K-3D monitors with 55-inch and 31-inch screens, respectively. The monitors can be positioned anywhere inside the operating room (OR) for the convenience of both surgeons and scrub nurses. Polarized 3D glasses are required to view the monitor images in 3D.

**Figure 4:**
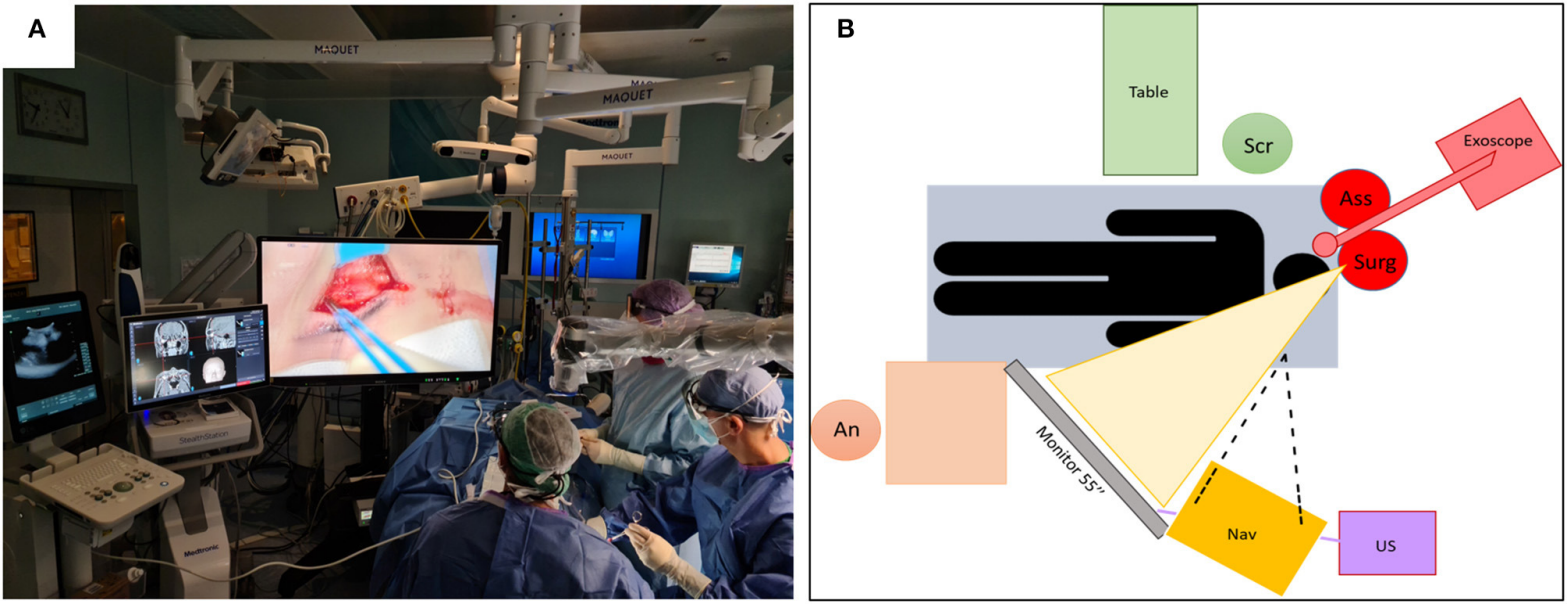
Placement of the technologies in use (4K-3D camera, 55-inch monitor, navigator, and ultrasound) and staff within the OR during surgery for right transpalpebral approach **(A)** and scheme **(B)**. Note the comfortable position of the surgeon who can operate while looking at the monitor with the head in an upright position.

For better understanding, the OR setting in our clinical case is shown in Figure 4.

## Discussion

It is widely accepted that symptomatic benign neoplasms of the orbit require treatment, with surgical resection as the treatment of choice for most CVMs. While the management of extraconic lesions can be easier, intraconic pathologies require a certain degree of expertise in manipulating the delicate muscle and nerve structures within the intraconic space.

The success of the procedure depends on the localization of the CVM in particular with respect to the optic nerve and the extrinsic musculature. For this reason, the choice of the surgical approach to be adopted depends not only on the site of the lesion, but also on the personal experience of the surgeon, always with the aim of less invasiveness and, consequently, of a better functional and esthetic result (3, 4).

It is thus clear that the best approach should ensure adequate visualization of the intraorbital muscular and neurovascular structures and the lesion, minimizing manipulation of the former.

Currently, the lateral aspect of the orbital content is generally approached *via* transcranial routes, such as fronto-orbitozygomatic, fronto-temporal, or supraorbital craniotomies, especially for lesions located posteriorly, and *via* lateral orbitotomy for more anterior ones (3, 4).

In recent years, the endoscopic transnasal approach to the orbital cavity has gained interest as a feasible and safe surgical technique for the management of CVMs located in the inferior medial and superior medial part of the orbit. In a review, Lenzi reported on 17 cases of CVMs, all located in the inferior medial part of the orbit, which were removed through an endonasal endoscopic approach, with few and minor complications except for a case of enophthalmos requiring surgical revision (12).

The advent of endoscopic approaches in orbital surgery has introduced the concept of less invasiveness, better esthetic result, and gain in terms of visualization. In this regard, Dallan et al. (13) emphasized how endoscopic visualization allows high magnification with angled view, if required, and with the light close to the structures, for better identification of anatomical details and, consequently, more precise surgery.

These authors concluded that new technologies, such as 3D screens, will be able to ensure even better visualization, and manipulation of orbital structures, working with the principles, more familiar to most surgeons, of microsurgery.

Over the years, transpalpebral and transconjunctival approaches, with or without osteotomies, have gained increasingly more space in orbital tumor and anterior skull base surgery due to their minimal invasiveness and optimal esthetic and functional outcomes (5, 13–15).

In case of CVMs located in the inferior lateral or superior lateral aspect of the intraconic space, the trans-eyelid approach is usually preferred, avoiding lateral or superior orbitotomy according to its position.

Zoli et al. recently performed a review of endonasal and transpalpebral endoscopic approaches for removal of orbital lesions, most of which were cavernous hemangiomas. They selected 23 lesions, of which 16, located medially, were removed by an endonasal approach and 7, located laterally, by a transpalpebral approach. In transpalpebral access, the use of an exoscope is reported in the initial part (i.e., skin incision) of the procedure and then abandoned in favor of the endoscope. The advantages of ultrasound in localizing the lesion within the orbit is also reported (5). In that publication, it was highlighted that knowledge of both endoscopic, endonasal, and transpalpebral techniques allows for 360° management of the anatomy of the orbit and thus the possibility to remove orbital lesions wherever they are located, whether lateral or medial, extraconic or intraconic.

Our expertise in transpalpebral approaches has grown over time thanks to the collaboration between neurosurgeons and maxillofacial surgeons, with the latter being more experienced in these approaches, mainly used by them in reconstructive surgery for orbital fractures. Experience to date suggests that trans-eyelid (superior or inferior) approaches provide adequate exposure of the entire orbital region, both medial and lateral, as well as inferior and superior.

In particular, as in the clinical case reported, an inferior transpalpebral approach offers complete extraconic exposure of the orbital floor up to the inferior third of the medial or lateral wall and posteriorly up to the inferior orbital fissure, enabling, by incision of periorbita, access to intraconic contents.

In daily surgical practice, where the endoscopic approach is not chosen, due to the surgeon's preference or lack of confidence with this technique, transpalpebral approaches are usually performed with visualization provided by loupes or operating microscope (OM). In recent years, exoscopes have been proposed as a possible alternative to the OM by offering very high magnification, high quality images, superior ergonomics, and ease of use. In the recent literature, different procedures have been performed with 4K-2D or 4K-3D exoscopic systems, in particular in plastic surgery as well as cranial, spine, and otologic surgery (6–11). A case of endoscopic dacryocystorhinostomy assisted by a 3D-2D exoscope has also been reported (16). These telescopes aim to combine the advantages of the OM with those of the endoscope, and at the same time, try to overcome the limits of both, such as the poor ease of movement and ability to adapt to the operating room setting, limits in the depth of field, and poor ergonomics for surgeons.

The high-resolution 4K-3D images of the orbital structures provided by the exoscope, starting with skin incision, make surgery more accurate, with optimal perception of depth, colors, and magnification. The high quality of the images, which allows precise manipulation of orbital structures, can be appreciated by watching the 4K-2D video of our case.

It should not be forgotten that the exoscope offers high quality, 4K-3D images not only for surgeons, but for everyone who watches the 55-inch or 31-inch monitor using polarized glasses. This advantage can also be used for educational purposes for students and residents, because the use of external monitors and glasses gives them the same high-resolution view as the surgeon.

An important disadvantage in using the exoscope is the difficulty in assisting the surgeon from a position of the assistant (5). This disadvantage is greatly reduced in our case, where the surgeon and the assistant worked side by side and therefore are able to look at the same 55-inch monitor placed in front of them, ensuring a precise manipulation for the assistant as well.

Moreover, the exoscope allows for neutral cervical spine posture by placing the monitor at eye level, directly in front of the surgeon, or at any angle desired. This ensures an upright and neutral posture, thus avoiding long hours spent in a fixed position looking through a microscope, or with the head bent over the patient wearing loupes.

We have no experience yet on the use of the exoscope in deep fields in orbital surgery. However, we do have extensive experience in this regard in intracranial surgery, e.g., for deep-seated lesions and with very angled surgical corridors, where the exoscope, compared to other visualization technologies and thanks to the possibility of easily tilting the camera, offers even in these cases the advantage of a clear view without forcing the surgeon to assume uncomfortable positions.

Furthermore, the exoscope allows more space around the operating table and patient. This is especially useful in procedures where multiple equipment is required, e.g., navigation devices or ultrasound, as in our surgical case.

The use of navigation allowed us to reduce the invasiveness of the procedure by addressing the tumor within the orbit. Even more, the choice of using ultrasound was driven by the desire to further reduce the margin of error before and just after periorbital incision due to the medial displacement of anatomical landmarks, and consequently poor navigation reliability, during ocular globe retraction. In fact, the use of a small size, high-frequency probe allowed us to limit even minimal errors of navigation, ensuring gentle manipulation of the delicate intraconic structures within periorbital fat.

In addition, throughout the time of hemangioma removal, the surgeon was able to look at the ultrasound images directly on the 55-inch monitor thanks to the Picture-in-Picture (PiP) mode offered by the exoscope (Figure 2). As reported by the surgeon, this was very useful because it allowed him to avoid frequently looking away from the operating field toward the ultrasound screen, reducing the risk of applying too much pressure on the ocular globe or minimal probe movement resulting in less accuracy in identifying the CVM within the intraconic space.

A slight difficulty in gaining a proprioceptive ability when manipulating the instruments by looking at the 3D monitor can be noticed by a surgeon who is unfamiliar with this technology. In our experience, the adaptation time is usually short and easily appreciable already during the same procedure. Indeed, as for all new technologies, a learning curve is required even for the exoscope.

In our opinion, the exoscope, by offering 4K-3D images with very high-definition and very high zoom, allows detailed anatomical visualization. This is very effective in procedures where high magnification is required, as in a minimal invasive approach to a small compartment such as the orbit. Moreover, the exoscope allows surgeons to operate in a comfortable position, even for extended periods, with all the images required in the same monitor. The use of navigation and ultrasound, which can be easily integrated with the exoscope thanks to the large space available above the operating field, makes the trans-eyelid approach even more minimally invasive, targeting surgical dissection, and consequently, minimizing manipulation of orbital intraconic structures, with optimal results for the patient from both functional and esthetic points of view.

## Data Availability Statement

The original contributions presented in the study are included in the article/Supplementary Material, further inquiries can be directed to the corresponding author/s.

## Ethics Statement

Ethical review and approval was not required for the study on human participants in accordance with the local legislation and institutional requirements. The patients/participants provided their written informed consent to participate in this study. Written informed consent was obtained from the individual(s) for the publication of any potentially identifiable images or data included in this article.

## Author Contributions

SPe and SPa designed the study, wrote the manuscript, and collected the data and images. RS reviewed the manuscript. All authors gave their final approval before submission, contributed to the article and approved the submitted version.

## Conflict of Interest

The authors declare that the research was conducted in the absence of any commercial or financial relationships that could be construed as a potential conflict of interest.

## Publisher's Note

All claims expressed in this article are solely those of the authors and do not necessarily represent those of their affiliated organizations, or those of the publisher, the editors and the reviewers. Any product that may be evaluated in this article, or claim that may be made by its manufacturer, is not guaranteed or endorsed by the publisher.

## Supplementary Material

Supplementary Video 1 Image definition and high magnification of anatomical details, as well as the use of navigation and ultrasound devices, can be clearly noticed and appreciated in this 4K-3D video.
